# Modeling Human Antitumor Responses *In Vivo* Using Umbilical Cord Blood-Engrafted Mice

**DOI:** 10.3389/fimmu.2018.00054

**Published:** 2018-01-26

**Authors:** Nicholas A. Zumwalde, Jenny E. Gumperz

**Affiliations:** ^1^Department of Medical Microbiology and Immunology, University of Wisconsin School of Medicine and Public Health, Madison, WI, United States

**Keywords:** humanized mice, umbilical cord blood, tumor immunotherapy, homeostatic proliferation, xenogeneic activation

## Abstract

Mice engrafted with human immune cells offer powerful *in vivo* model systems to investigate molecular and cellular processes of tumorigenesis, as well as to test therapeutic approaches to treat the resulting cancer. The use of umbilical cord blood mononuclear cells as a source of human immune cells for engraftment is technically straightforward, and provides T lymphocytes and autologous antigen-presenting cells (including B cells, monocytes, and DCs) that bear cognate antigen presenting molecules. By using a human-specific oncogenic virus, such as Epstein-Barr virus, *de novo* neoplastic transformation of the human B cells can be induced *in vivo* in a manner that models progressive stages of tumorigenesis from nascent neoplasia to the establishment of vascularized tumor masses with an immunosuppressive environment. Moreover, since tumorigenesis occurs in the presence of autologous T cells, this type of system can be used to investigate how T cells become suppressed during tumorigenesis, and how immunotherapies counteract immunosuppression. This minireview will provide a brief overview of the use of human umbilical cord blood transplanted into immunodeficient murine hosts to model antitumor responses.

## Introduction

While animal model systems, and particularly laboratory mouse strains, are absolutely indispensible for understanding the basic biology of both cancers and the immune system, preclinical analyses of tumor immunotherapy are also likely to benefit from experimental systems that utilize primary human cells obtained from genetically diverse individuals. In this minireview, we will discuss the use of immune-deficient mice engrafted with human umbilical cord blood cells for studying human T cell biology and tumor immunotherapy *in vivo*.

Clinical applications of tumor immunotherapy currently center on two main approaches. The first is the use of “checkpoint” blockade antibodies to relieve PD-1 and CTLA-4 mediated immunosuppression of endogenous T cells. When these inhibitory pathways are disabled by blocking antibodies, a patient’s existing T cells can often induce tumor regression (1, 2). The second approach, cellular immunotherapy, involves administering cytolytic lymphocytes that have been expanded *in vitro*, and act as direct antitumor effectors within the patient. Most prominent in this category is the use of chimeric antigen receptor (CAR) T cells that have been genetically modified to specifically target the patient’s tumor (3). Refining and further developing tumor immunotherapeutic approaches will require experimental model systems that allow us to better understand interactions between human immune effectors and human tumors *in vivo*. In particular, it would be helpful to be able to model human T cell functions during progressive stages of tumorigenesis, from nascent neoplasia to the establishment of tumors with an immunosuppressive environment. Also key is to be able to assess *in vivo* responses of human T cells that are autologous to the tumor (e.g., those targeted by checkpoint blockade), as well as to test the impact of exogenously administered effectors (e.g., CAR-T cells) on established tumors. These elements are provided by new experimental models in which immunodeficient mice are engrafted with human immune cells, and human tumor formation is induced *in vivo via* infection with an oncogenic virus.

## Engraftment of Mice with Human Immune Cells

### Mouse Strains

Adoptive transfer of human immune cells into murine hosts is most successful in mouse strains lacking adaptive immune cells that also have impairments in innate cell types, such as NK cells, that would otherwise kill engrafted human cells. One strain that is now commonly used for human cell engraftment is the NOD-SCID-Gamma or “NSG” mouse (NOD.Cg-Prkdc^scid^Il2rg*^tm1Wjl^*/SzJ). NSG mice fail to develop T and B cells due to the *Prkdc^scid^* mutation, are defective in multiple innate immune functions because they are bred onto a NOD background and are also knocked out for the common γ chain of the IL-2 receptor, which is required for proper development of multiple lineages, including NK cells (4). The NSG strain shows little or no evidence of “leakiness” in regards to development of murine T cells, has highly deficient murine NK cells, and has been found to provide an excellent environment for the survival of human cells *in vivo* (5). Building on the utility of the NSG strain, strains with further genetic modifications have been generated that show additional improvements in human cell engraftment. These include strains that are transgenic for key human cytokines that promote hematopoiesis (e.g., TPO, CSF1, IL3, CSF2), and a strain lacking c-Kit that supports high levels of human hematopoietic engraftment without irradiation or myeloablative conditioning (6–10).

### Hematopoietic Stem Cell (HSC) Engraftment

**Figure 1:**
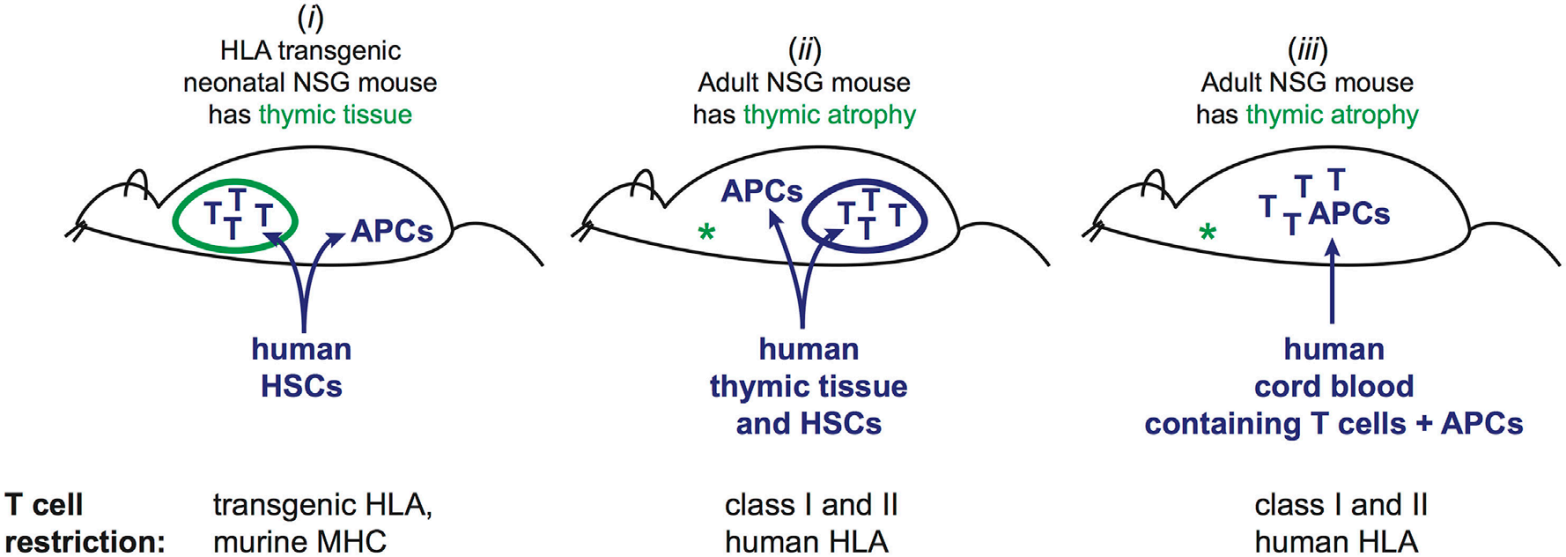
Three different approaches to generate mice engrafted with human T cells and cognate human antigen-presenting cells (APCs). (i) Injection of human hematopoietic stem cells (HSCs) into neonatal mice. Human T cells and APCs develop from the HSCs. T cells undergo selection in murine thymus based on interactions with murine cells. By using mice that are transgenic for one or more HLA molecules, and HSCs bearing HLA alleles that match the transgenes, a fraction of the mature human T cells in the periphery will be able to recognize the human APCs, while others are restricted by murine antigen presenting molecules that are also present in the thymic environment. This method does not recapitulate the full repertoire of T cell restriction for human antigen presenting and may not produce tolerance to human peptides presented by the restricting HLA molecules, but is associated with little or no graft-versus-host disease (GVHD). (ii) Adult NSG mice, which lack murine thymic tissue due to atrophy, are injected with human HSCs. Concurrently, a fragment of human thymic tissue is surgically implanted. Human T cells and APCs develop from the HSCs, and the thymic fragment develops into a viable organoid. T cells undergo selection in the human thymic organoid based on interactions with human thymic cells. The resulting T cell repertoire includes restriction for the full panoply of autologous HLA molecules. However, signs of chronic GVHD typically manifest within 4–6 months. (iii) Human umbilical cord blood engraftment of adult NSG mice. Mature T cells (selected in the baby’s thymus) are transplanted along with autologous APCs. Human T cells typically persist for at least 3 months, but signs of GVHD may become apparent after about 2 months.

Engraftment of human immune cells into mice can be successfully accomplished through a variety of different protocols. However, different approaches entail key differences in the selection and specificity of the human T cell compartment that is then present in the engrafted mice. A central distinction is whether human HSCs are used to give rise to T cells that develop within the murine host, or whether T cells that have already undergone selection in the human donor are transferred into the mice (Figure 1). NSG mice possess thymic tissue at birth, but this tissue normally atrophies due to the absence of murine T cells, and becomes essentially undetectable within 6 weeks after birth. Engraftment protocols that transfer human HSCs into neonatal mice result in colonization of the murine thymus by human pre-T cells, which promotes the survival of the thymic tissue, and provides an environment for selection of the human T cells (11). Because the human T cells develop within the murine thymus, they undergo positive and negative selection on murine antigen presenting molecules. As a result, tolerance to murine tissues is established, but the T cells are not optimized for interactions with human antigen-presenting cells (APCs) that also develop from the engrafted HSCs. However, by instead using mice that are transgenic for one or more human HLA molecules, some of the human T cells that are generated are able to interact productively with human APCs (12). Nevertheless, a potential drawback is that many of the human T cells will be developmentally selected on murine antigen presenting molecules (Figure 1, part *i*), and thus the human T cell compartment probably does not fully recapitulate the specificities and lineages of human T cells.

### Human Thymic Engraftment

An alternative method is to transfer human HSCs into mice at 6–8 weeks of age (when the murine thymus is gone), and to cotransplant fragments of human thymic tissue, which are typically surgically implanted under the kidney capsule. This results in the growth of a human thymic organoid within the mice that allows for T cell selection by human thymic epithelial cells, and generates a T cell repertoire specific for the full complement of human HLA molecules (Figure 1, part *ii*). This approach has been shown to enable productive interactions of human T cells with autologous human APCs that also develop in the murine host from human HSCs (13–15). We have shown that this approach results in the generation of human T cells that recognize human non-classical antigen presenting molecules, such as CD1 molecules, and thus enables modeling of select T cell populations that are present in humans but not found in mice (16). Central disadvantages of this type of approach are the challenges associated with implanting human thymic tissue in the mice, and the length of time required for full establishment of the human immune compartment in the periphery, which typically requires about 3 months after tissue engraftment. An additional concern is that signs of graft-versus-host disease (GVHD) often become apparent within about 4–5 months after tissue engraftment (17).

### Engraftment of Mature Lymphocytes

An alternative that addresses some of the challenges of the above approaches is to transfer mature human immune cells into NSG mice. While transferring adult human PBMCs into immune-deficient mice typically results in acute GVHD pathology that manifests within 3–6 weeks (17), it is nevertheless possible to model functional interactions amongst populations of human immune cells in a short-term manner using cells from adults. For example, inflammatory responses induced by interactions among human immune cells can be read-out after 24–48 h using a vascularized peripheral tissue of the mouse, such as the footpad (18, 19). Alternatively, adult human PBMCs can be systemically transferred into immune-deficient mice for short periods to investigate functional capabilities of specific populations of human lymphocytes. For example, studies of this type have demonstrated that human Vγ9^+^Vδ2^+^ T cells can be sufficient to control the outgrowth of xenografted human tumors (20–25). Nevertheless, while adoptive transfer of immune cells from human adults into NSG mice provides an important means of investigating functional interactions of human cells *in vivo*, the GVHD responses associated with this approach significantly limit investigation of longer-term immunological processes.

In contrast, adoptive transfer of NSG mice with human umbilical cord blood mononuclear cells (CBMCs) provides a means of modeling human immune interactions *in vivo* over a longer period of time. CBMCs contain mature human T cells that were selected in a fully human environment (i.e., the baby), and that are appropriately restricted for the accompanying human APCs (e.g., B cells, monocytes, DCs), but that are as yet in a highly naive state. By removing the CD34^+^ HSCs prior to transplantation, new human T cells will not develop after transfer, and thus the mice contain only the T cells that were selected in a human thymus and that are restricted by the antigen presenting molecules expressed on the autologous APCs that were cotransferred in the CBMC sample (Figure 1, part *iii*). The adoptively transferred human T cells typically expand and persist in the mice for at least 2 months without evidence of significant GVHD pathology, which provides an experimental window that is adequate for many types of analyses. As discussed below, the central concern about this approach relates to the functional competence of the cord blood T cells after engraftment.

## Functional Characteristics of Human Cord Blood T Cells

As evidenced by their expression of CD45RA and not CD45RO, cord blood T cells are naive (26), and thus they would be expected to show less efficient cytokine production compared to previously activated T cells. However, a number of observations suggest that cord T cells may also be less functionally competent than naive peripheral blood T cells that are found later in life. Exposure to IL-10 produced by trophoblasts suppresses placental T cell activity, and the hormonal environment of pregnancy may also dampen T cell activity (27–29). It is not clear how long after birth these suppressive effects last, however, cord blood has been found to contain only very low percentages of T cells capable of producing IL-2, IFNγ, TNFα, and IL-4 (30, 31). Cord T cells also lack the constitutive expression of perforin seen in adult CD8^+^ T cells (32). The inefficient effector cytokine production of cord T cells may be due to epigenetic alterations, since cord blood CD4^+^ T cells were found to have hypermethylation of the IFNγ promoter (33). Additionally, PKCζ expression levels appeared to be reduced in neonatal T cells, which correlated with a deficiency in IFNγ production and affected their ability to mature into effector cytokine producing cells (34, 35). Perhaps as a result of these features, cord blood T cells are associated with substantially reduced incidence of GVHD following hematopoietic transplantation (31, 36).

Nevertheless, effector T cell responses to pathogens do occur early in life (29), indicating that cord blood T cells are capable of becoming functionally activated. Moreover, it has recently been shown that the gene expression differences that distinguish specific-pathogen-free mice from “wild” mice (i.e., those that have experienced microbial exposure found in the natural world) closely resembled the differences between human umbilical cord blood and adult peripheral blood cells (37). Thus, many of the functional characteristics of cord blood T cells may be due to a lack of immunological experience, rather than to features that have a lasting effect on gene expression. Consistent with this, it is now clear that cord blood T cells, like naive T cells from adults, can readily be activated to undergo expansion, maturation, and polarization.

## Polarization of Cord T Cells into Effectors

T cells derived from cord blood can be expanded *in vitro* by anti-CD3 and anti-CD28 antibody stimulation in the presence of IL-2 (38). While such antibody-driven expansion of cord T cells *in vitro* is associated with significant apoptosis, this is mitigated in the presence of IL-7, which also aids in maintaining higher T cell receptor (TCR) Vβ diversity (39). When IL-12 is present during CD3/CD28 stimulation, the cord T cells rapidly acquire Th1-polarization features such as the ability to produce IFNγ, TNFα, and granzyme A (38), and subsequently show enhanced IFNγ production after TCR stimulation suggesting a lasting polarization toward a Th1 phenotype (40). Conversely, exposing cord T cells to IL-4 during CD3/CD28 stimulation leads to Th2-skewing (40). However, this occurs more slowly and requires repeated TCR stimulation in the presence of IL-4 in order to maintain production of IL-10, IL-4, IL-5, and IL-13. Hence, cord cells that have been Th2-skewed by short-term IL-4 exposure maintain the plasticity to revert back to a Th0 phenotype, and can even convert to a Th1 profile with the addition of IL-12, suggesting that they are not intrinsically biased toward a Th2 phenotype (40). These results illustrate that, despite their initial functional reticence, the effector capabilities of cord-derived T lymphocytes do not remain suppressed in the long-term.

## Events after Transfer into Immune-Deficient Mice

**Figure 2:**
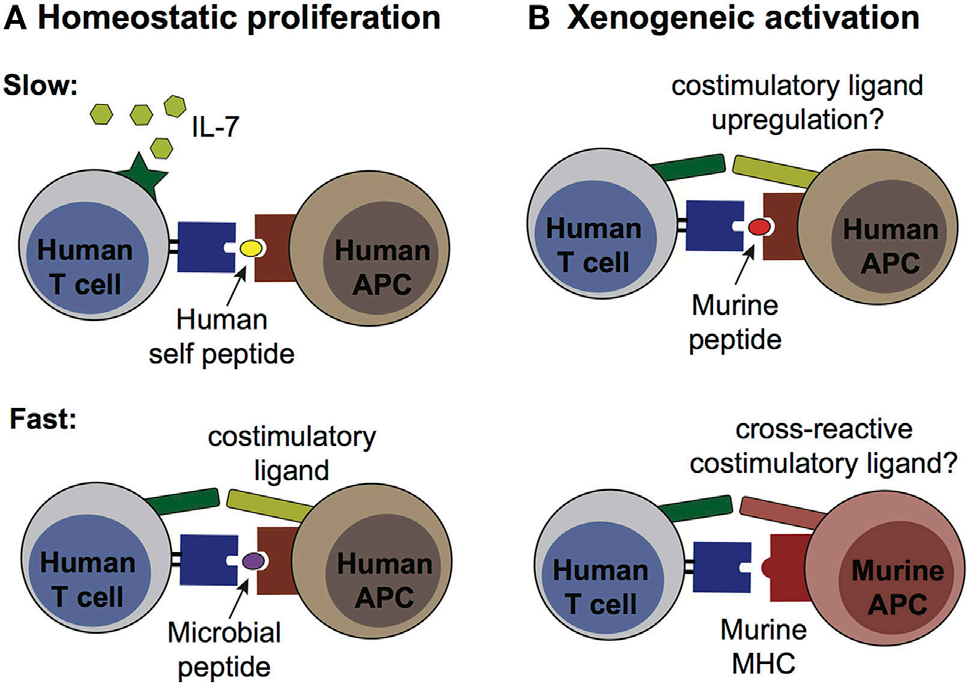
Pathways of human T cell activation in murine engraftment models. **(A)** Homeostatic proliferation occurs via two distinct processes, termed “slow” and “fast.” Slow proliferation (top panel) results from TCR stimulation by a weak agonist [e.g., autologous antigen-presenting cells (APCs) bearing self peptides] in the presence of IL-7, and does not require co-stimulatory ligands. Since murine IL-7 is recognized by human IL-7 receptors, this cytokine is likely to have high availability after transplantation into NSG mice. This pathway likely affects most of the transplanted human T cells. Fast proliferation (bottom panel) is driven by T cell receptor (TCR) recognition of high affinity antigens (e.g., microbial peptides from commensal species) in the presence of co-stimulatory ligands, and does not require IL-7. This process would likely only affect a subset of the transplanted T cells. **(B)** Xenogeneic activation may result from T cell recognition of murine peptides presented by self HLA molecules on human APCs (top panel), or by cross-reactivity of human TCRs for murine MHC molecules on murine APCs (bottom panel). Upregulation of co-stimulatory ligands by the human APCs or expression of cross-reactive co-stimulatory ligands by the murine APCs might be required for this pathway to induce productive activation, rather than anergy (which would be expected from TCR stimulation in the absence of co-stimulation). Xenogeneic activation pathways would be expected to affect only a subset of the transplanted human T cells.

Since transfer of human cord T cells into murine hosts is likely to be associated with exposure to stimulating factors, it is important to consider the impact this might have on the subsequent functionality of the T cells. Major factors that might cause T cell activation after transfer include the lymphopenic environment of the host and exposure to xenogeneic antigens (see Figure 2). Whereas activation resulting from xenoantigenic stimulation might be an artifact of transferring human T cells into a murine host, T cell activation that is due to a lymphopenic environment is a process that occurs physiologically (41). Thus, changes in the human cord T cell population following transplantation into mice are not necessarily an indication of aberrant activation due to the use of a xenogeneic model system. Indeed, cord blood transplantation in human patients also results in thymic independent expansion of the transplanted T cells that is associated with a rapid shift from a naïve to memory phenotype (42). Hence, the lymphopenic environment of NSG mice might be expected to similarly induce a proliferative response from adoptively transferred cord T cells and might affect the nature of the TCR repertoire.

The spontaneous cellular division induced in a lymphopenic environment is broadly delineated into two major varieties: slow and fast. Slow homeostatic proliferation is driven by T cell recognition of low affinity peptides (e.g., self peptides) bound to MHC in the presence of IL-7 (43, 44). This slow spontaneous division proceeds in the absence of costimulation by CD28, CD40, or LFA-1, and appears to largely occur within T cell zones in secondary lymphoid organs (44). In contrast, fast homeostatic proliferation is an antigen driven response from a smaller subset of T cells and is IL-7 independent (44). This pathway requires costimulatory signals and leads to swift upregulation of memory markers and acquisition of effector functions such as the production of IL-2 and IFNγ. Since fast homeostatic proliferation is greatly reduced when recipient mice are housed in germ-free conditions, it has been proposed that peptides from gut microbes are a major driver of this response (44).

By engrafting NSG mice with total CBMCs, APCs bearing cognate antigen presenting molecules are present that could activate either fast or slow homeostatic proliferation by the cord T cells. However, since NSG mice are typically housed in aseptic conditions, the supply of microbial peptides required for fast homeostatic proliferation might be limited. In contrast, since murine IL-7 is highly cross-reactive with the IL-7 receptors of human cells (45), the conditions for slow homeostatic proliferation are likely to be present (Figure 2A). Thus, most or all human cord T cells are expected to proliferate homeostatically after transplantation into NSG mice, although it remains unclear whether this process substantially alters their functional state.

The second major factor that might affect the functional status of transplanted cord T cells is xenoantigenic stimulation. This might occur either *via* TCR binding to murine MHC molecules or *via* recognition of murine peptides presented by self HLA molecules on other human cells (Figure 2B). In either case, such xenoantigenic activation would be predicted to affect only the subset of the cord T cells bearing a cross-reactive TCR. The strength of TCR signaling delivered by these recognition events is difficult to predict, and it is also not clear whether TCR signals of this type are typically accompanied by costimulatory signals that are required for a productive antigen-driven response. In the absence of appropriate costimulation xenoantigen-associated TCR signals would be expected to induce anergy, whereas if costimulation is present a classic antigen-driven expansion of the responding T cells would be expected. Importantly, productive xenoantigenic activation of cord T cells within murine hosts would be expected to result in GVHD. However, there is typically little evidence of GVHD within the first 2 months after administering cord T cells (Zumwalde and Gumperz unpublished data). Thus, xenoantigenic activation may not play a major role for a period of weeks or months after the adoptive transfer of human cord blood T cells into NSG mice.

## Modeling Tumor Immunity

Perhaps surprisingly, given the evidence suggesting their effector functions are limited, cord T cells have been found to be capable of mediating efficient antitumor responses. Analyses of cord T cells exposed to tumor cells *in vitro* have unambiguously established that they can mount both cytotoxic and cytokine responses (46–49). Moreover, the ability of cord T cells to carry out antitumor effects *in vivo* has been clearly demonstrated in recent studies (47, 50). In a particularly revealing analysis, T cells purified from cord or adult blood were compared in a head-to-head manner for their ability to limit the growth of allogeneic EBV-transformed B cells (EBV-LCLs) in NSG mice (51). The EBV-LCLs were injected subcutaneously into NSG mice, leading to the formation of a solid mass at the site of injection resembling a tumor. Equivalent doses of primary cord or adult T cells were injected intravenously, and growth of the EBV-LCL mass was monitored. Administration of cord T cells was associated with significantly less growth of the implanted EBV-LCLs compared to adult T cells, and the EBV-LCL masses also showed substantially more evidence of T cell infiltration in the cord T cell treatment group (51). Studies such as these demonstrate that cord T cells are capable of promoting tumor rejection *in vivo*. However, such experimental systems, where *in vitro*-derived tumor cells are administered to immunodeficient mice and cytolytic lymphocytes are then added to test for tumor rejection, do not clearly model the impact of immunosuppressive tumor environments that must be overcome by successful immunotherapeutic strategies.

To address this need, we have developed an experimental model in which neoplastic transformation of human B cells occurs *in vivo* and is associated with suppression of the endogenous T cells. Briefly, NSG mice are injected with CD34-depleted CBMCs in the presence of a lytic strain of EBV, called M81, that was recently isolated from a patient (52). The virus induces *de novo* neoplastic transformation of the initially healthy B cells, and within approximately 4 weeks nearly all of the mice typically develop large tumors (53). The lymphomas are located in the peritoneal cavity adjacent to pancreas, liver, or bile ducts, and ultimately end up invading nearby organ tissue and causing mortality. The lymphomas in this model are heavily infiltrated by autologous CD4^+^ and CD8^+^ T cells, but the tumor B cells express elevated levels of the inhibitory ligands PD-L1 and PD-L2 (54). Antibody-mediated blockade of PD-1 and CTLA-4 results in reduced tumor burden, prolongs survival, and reveals EBV peptide-specific T cell responses by the autologous T cells (54). Thus, lymphoma-specific T cells are generated in this model, but are usually held in check by suppressive pathways.

We have recently shown that cellular immunotherapy using human γδ T cells has potent antitumor immune effects in this model both at early stages of nascent neoplasia (immunosurveillance) and at later stages after solid tumors containing immunosuppressive ligands have become established (55). Hence, even in the absence of checkpoint blockade, the immunotherapeutic γδ T cells are apparently able to overcome the immunosuppressive environment that stymies the responses of the endogenous T cells. We expect that preclinical models of this type will provide a valuable tool to investigate molecular and cellular mechanisms by which successful immunotherapeutic strategies overcome or avoid suppressive environments created by tumors.

## Author Contributions

NZ reviewed literature and cowrote manuscript; JG generated figures and cowrote the manuscript.

## Conflict of Interest Statement

The authors declare that the research was conducted in the absence of any commercial or financial relationships that could be construed as a potential conflict of interest.
